# Boosting-based ensemble machine learning models for predicting unconfined compressive strength of geopolymer stabilized clayey soil

**DOI:** 10.1038/s41598-024-52825-7

**Published:** 2024-01-28

**Authors:** Gamil M. S. Abdullah, Mahmood Ahmad, Muhammad Babur, Muhammad Usman Badshah, Ramez A. Al-Mansob, Yaser Gamil, Muhammad Fawad

**Affiliations:** 1 Department of Civil Engineering, College of Engineering, Najran University, P.O. 1988, Najran, Saudi Arabia; 2 Institute of Energy Infrastructure, Universiti Tenaga Nasional, Kajang, 43000 Malaysia; 3 Department of Civil Engineering, University of Engineering and Technology Peshawar (Bannu Campus), Bannu, 28100 Pakistan; 4 Department of Civil Engineering, Faculty of Engineering, University of Central Punjab, Lahore, 54000 Pakistan; 5 Water Wing, Water and Power Development Authority (WAPDA), WAPDA House Peshawar, Peshawar, 25000 Pakistan; 6 Department of Civil Engineering, Faculty of Engineering, International Islamic University Malaysia, Jalan Gombak, Selangor, 50728 Malaysia; 7 Department of Civil, Environmental and Natural Resources Engineering, Luleå University of Technology, Luleå, Sweden; 8 Department of Civil Engineering, School of Engineering, Monash University Malaysia, Jalan Lagoon Selatan, Bandar Sunway, 47500 Selangor Malaysia; 9 Silesian University of Technology, Gliwice, Poland; 10 Budapest University of Technology and Economics, Budapest, Hungary

**Keywords:** Civil engineering, Materials science

## Abstract

The present research employs new boosting-based ensemble machine learning models i.e., gradient boosting (GB) and adaptive boosting (AdaBoost) to predict the unconfined compressive strength (UCS) of geopolymer stabilized clayey soil. The GB and AdaBoost models were developed and validated using 270 clayey soil samples stabilized with geopolymer, with ground-granulated blast-furnace slag and fly ash as source materials and sodium hydroxide solution as alkali activator. The database was randomly divided into training (80%) and testing (20%) sets for model development and validation. Several performance metrics, including coefficient of determination (R^2^), mean absolute error (MAE), root mean square error (RMSE), and mean squared error (MSE), were utilized to assess the accuracy and reliability of the developed models. The statistical results of this research showed that the GB and AdaBoost are reliable models based on the obtained values of R^2^ (= 0.980, 0.975), MAE (= 0.585, 0.655), RMSE (= 0.969, 1.088), and MSE (= 0.940, 1.185) for the testing dataset, respectively compared to the widely used artificial neural network, random forest, extreme gradient boosting, multivariable regression, and multi-gen genetic programming based models. Furthermore, the sensitivity analysis result shows that ground-granulated blast-furnace slag content was the key parameter affecting the UCS.

## Introduction

The study of geopolymer technology for improving cohesive soil properties is significant from a scientific and practical perspective. The utilization of stabilizers such as lime and fly ash has been employed to improve the geotechnical properties of cohesive soils. The term "geopolymer" is commonly used to describe inorganic substances that are produced by the synthesis of aluminosilicate compounds. The raw material system utilized in the production of geopolymer materials comprises two primary constituents, namely, starting materials and alkaline chemical active ingredients. The investigation of geopolymer materials is being conducted with the aim of developing environmentally sustainable products made from industrial waste materials that possess significant utility^1–3^. Geopolymer has a wide range of applications in acid-resistant cement, unburned masonry, quick-setting cement, and fireproof materials. Geopolymer technology is a subject of considerable interest and extensive research globally, exhibiting promising prospects for further development. Geopolymer has emerged as a viable alternative to standard Portland cement due to its composition as a synthetic alkali aluminosilicate material. This material is created through the reaction of solid aluminosilicate with a solution containing a combination of hydroxide-silicate or a concentrated aqueous alkali hydroxide^4,5^. its production process uses less fuel energy overall and produces less greenhouse gas emissions overall^6,7^. Geopolymers can be synthesized with a solid aluminosilicate material obtained from diverse sources of industrial waste, such as silicate and/or alumina components. The acronyms for these materials include ground-granulated blast-furnace slag (S), metakaolin, and fly ash (FA)^8,9^. In geotechnical engineering projects, FA or S has been utilized for soil improvement^10,11^. Building foundations, highways, dams, canals, and other similar constructions are examples of embankment works^12–14^. Based on previous research, the introduction of S or FA into the soil has been found to potentially improve its mechanical strength^10,15–17^. Sharma and Sivapullaiah conducted a study to investigate the efficacy of FA and S in soil stabilization applications^18^. The curing durations of 7, 14, and 28 days were used to examine the properties of S and FA. The stabilized soil's strength of 0.45 MPa was obtained after 28 days of curing, and its plastic limit and water content values were both decreased. Based on the findings, the utilization of S and FA as binders presents a novel prospect for enhancing the activity of pozzolans, hence potentially increasing the unconfined compressive strength (UCS) and reducing the swelling potential of clay soils^15–17,19^. Many researchers undertook work on soil stabilization using different materials e.g.^20–22^. Abdullah and Shahin^23^ investigated the geo-mechanical characteristics of a clay–binder composite that incorporates a unique mix of fly ash activated by alkali. This combination leads to the formation of a geopolymer material that exhibits cement-like qualities upon hardening in soil. The findings indicate that geopolymer-treated clay specimens exhibit superior mechanical properties compared to untreated clay specimens, as demonstrated by the results of the UCS test and consolidated undrained (CU) test. Depending on the amount of geopolymer and curing time, the unconfined compressive strength could increase by up to six times. Rios et al.^24^ carried a total of 16 specimens of geopolymer-stabilized soil tests to analyze unconfined compressive strength (UCS) and stiffness. These specimens were prepared using different quantities of fly ash, soil, and alkaline solutions.

In general, determining the geotechnical parameters of soft soil is a laborious, time-consuming, costly, and energy-intensive procedure that requires a great deal of effort, equipment, and time. In order to acquire precise data pertaining to the compaction characteristics and UCS of soils, a minimum of six tests and four tests, respectively, must be conducted^9^.

In order to accurately predict compaction parameters, UCS, and other soil properties, predictive models have been developed. These methodologies often result in the development of equations including several undetermined coefficients, which may have an influence on the relationships between independent and dependent variables. In spite of being successful in some scenarios of stabilized soils, the resulting models are intrinsically erroneous despite, primarily due to their complexity. Machine learning (ML) models have been used successfully in various domains such as in geotechnical issues including prediction of the UCS^25–41^. Boosting is a ML technique that leverages the strengths of ensemble methods, which involve combining multiple weak learners to construct a powerful and effective learner. The utilization of this approach enhances the efficiency of following learning models, concurrently reducing the errors associated with the preceding learning models. There are various types of boosting algorithms, two of which are gradient boosting (GB) and adaptive boosting (AdaBoost)^42^.

A comprehensive literature review reveals that GB and AdaBoost ML models have not yet been used to predict the UCS of clayey soil stabilized with geopolymer, despite the fact that these are the most effective and widely used ML methods. Therefore, an attempt was made to evaluate its capability for predicting the UCS of clayey soil stabilized with geopolymer, while bearing in mind the applicability of this modeling approach in civil engineering applications. This study focuses on development of boosting-based ML models to assess the potential use of these models in rapidly predicting the UCS. The dataset of total 270 soil samples consists of a single dependent variable, the UCS, and several independent variables including the ground-granulated blast-furnace slag (S; %), the plasticity index (PI; %), the alkali-to-binder ratios (A/B), the percentage of fly ash (FA; %), the molar concentrations of an alkali solution (M; mol/l), and the ratios of Si/Al and Na/Al. The evaluation of the model's performance was conducted using four performance metrics, namely mean absolute error (MAE), root mean square error (RMSE), mean squared error (MSE), and coefficient of determination (R^2^). The main contributions of this study are:

1. To investigate the feasibility of boosting-based ensemble ML models for predicting UCS of geopolymer stabilized clayey soil and to provide executable models for frequent use in practice;
2. To compare the performance of boosting-based ensemble ML models, such as GB and AdaBoost, with that of some of the most widely used ML models; and
3. To investigate the relative importance of the factors influencing the UCS of geopolymer stabilized clayey soil using a sensitivity analysis.

## Related literature

Recently, several ML models have been proposed to estimate the UCS of geopolymer stabilized soil (see Table 1) such as artificial neural network (ANN), support vector machine (SVM), random forest (RF), multi-gen genetic programming (MGGP), hybrid neuro-fuzzy (NF)-group method of data handling (GMDH) and particle swarm optimization (PSO). For example, Mozumder and Laskar^43^ utilized a database including 282 samples and 8 input factors, namely liquid limit (LL), plastic index (PI), ground-granulated blast-furnace slag content (S), fly ash content (FA), molarity of sodium hydroxide (NaOH) concentration (M), alkaline content to binder content ratio (A/B), atomic number ratio of sodium to aluminum (Na/Al), and atomic number ratio of silicon to aluminum (Si/Al). The input parameters used in this study were employed for the development of an ANN model, specifically a multi-layer perception (MLP) feed-forward network, with the Bayesian Regularization back propagation training technique. The objective of this model was to predict the UCS of clayey soil stabilized with geopolymer. The ANN model, as described by Mozumder and Laskar^43^ demonstrates a high level of performance in the testing dataset as indicated by the coefficient of determination (R^2^ = 0.9643). Mozumder et al.^44^ developed a new dataset with the same input variables excluding FA content from the database of Mozumder and Laskar^43^. Mozumder et al.^44^ used SVM to predict the UCS of clayey soil stabilized with geopolymer having R^2^ = 0.9801 for testing dataset. Soleimani et al.^8^ created a MGGP model to estimate the UCS of geopolymer stabilized soil. The model utilized an original database consisting of 282 samples and 8 input variables. The model exhibited strong performance, as evidenced by an R^2^ value of 0.9420 and MAE of 1.071 MPa when evaluated on the testing dataset. Javdanian and Lee^45^ developed a hybrid ML model that combines NF, GMDH and PSO, known as NF-GMDH-PSO was devised to predict the UCS of stabilized cohesive soils using geopolymers. The model's performance was evaluated using the following metrics: R^2^ = 0.971, mean absolute error (MAE) = 0.231 MPa, and root mean square error (RMSE) = 0.401 MPa. Nagaraju and Prasad^46^ investigated the efficacy of the PSO technique in predicting the UCS of geopolymer-stabilized expansive blended clays. Zeini et al.^9^ utilized random forest (RF) algorithm to predict the UCS of geopolymer stabilized clayey soil. The researchers employed the primary database and assessed the efficacy of the machine learning model by analyzing the testing dataset. This analysis resulted with the R^2^ value of 0.9757 and the RMSE value of 0.9815 MPa. ANN is used by Ngo et al.^47^ to predict the UCS of geopolymer stabilized clayey soil and found reliable results with R^2^ = 0.9808, RMSE = 0.8808 MPa, and MAE = 0.6344 MPa. Despite the fact that the aforementioned models can predict the UCS, there is still room for improvement in terms of accuracy. Therefore, this field is still being researched and investigated.

**Table 1:** Summary of recent advances in predicting unconfined compressive strength of geopolymer stabilized clayey soil.

Model	Input parameter	Data size	Performance metric on test set	References
ANN	LL, PI, GGBFS, FA, M, A/B Na/Al, Si/Al	282	R^2^ = 0.9643	Mozumder and Laskar^43^
SVM	LL, PI, GGBFS, M, A/B Na/Al, Si/Al	213	R^2^ = 0.9801	Mozumder et al.^44^
MGGP	LL, PI, GGBFS, FA, M, A/B Na/Al, Si/Al	282	R^2^ = 0.9420 MAE = 1.0710 MPa	Soleimani et al.^8^
NF-GMDH-PSO	LL, PI, GGBFS, FA, M, A/B Na/Al, Si/Al	282	R^2^ = 0.9710 RMSE = 0.4010 MPa MAE = 0.2310 MPa	Javdanian and Lee^45^
RF	LL, PI, GGBFS, FA, M, A/B Na/Al, Si/Al	282	R^2^ = 0.9757 RMSE = 0.9815 MPa	Zeini et al.^9^
ANN	LL, GGBFS, FA, M, A/B Na/Al, Si/Al	282	R^2^ = 0.9808 RMSE = 0.8808 MPa MAE = 0.6344 MPa	Ngo et al.^47^

## Dataset and correlation analysis

The present study utilized a database that was obtained from the study conducted by Mozumder and Laskar^43^. A total of 270 unconfined compressive strength (UCS) samples of cohesive soils stabilized with geopolymer has been collected (see Supplementary File, Appendix A, Table A1). The experiments were conducted on three types of cohesive soils. Fly ash (FA) and ground-granulated blast-furnace slag (S) as well as their combinations were used as the source materials for geopolymerization^43^.

The amount of alkali to binder proportion (A/B), molar concentration of alkali solution (M), atomic proportions of silicon to aluminum (Si/Al), and sodium to aluminum (Na/Al) were varied in the experiments and the influence of these parameters on the unconfined compressive strength (UCS) of stabilized cohesive soils was investigated^43^. Hence, the parameters plasticity index (PI; %), liquid limit (LL; %), ground-granulated blast-furnace slag (S; %), fly ash (FA; %), A/B, M (moles per liter (mol/l)), Si/ AL, and Na/Al were considered as inputs parameters. In addition, the USC parameter was considered as output in the model development. The binder content exhibited a range of 4–50% for S, 4–20% for FA, and a composite of S and FA, denoted as a ratio relative to the dry weight of the soil solids. In the investigation of Mozumder and Laskar^43^, alkali solutions with molar concentrations of 4 M, 8 M, 10 M, 12 M, and 14.5 M were used in the experiment. The weight ratio of the alkali solution to the binder (A/B) was selected as 0.45, 0.65, and 0.85. The 28 day UCS test on the samples was carried out in line with Indian Standard: 2720^48^. The 270-sample dataset, which contains various UCS tests, is summarized in Table 2, and the inputs and output statistics of the present study are shown in Table 3.

**Table 2:** The present study's inputs and outputs.

S. No	LL (%)	PI (%)	S (%)	FA (%)	M (mol/l)	A/B	Na/Al	Si/Al	UCS (MPa)
1	116	88.46	20	0	4	0.45	0.39	1.49	0.0595
2	116	88.46	16	0	4	0.45	0.39	1.49	0.0616
3	116	88.46	12	0	4	0.45	0.39	1.49	0.0551
…	…	…	…	…	…	…	…	…	…
…	…	…	…	…	…	…	…	…	…
…	…	…	…	…	…	…	…	…	…
268	38	14.07	0	16	12	0.65	1.18	2.49	0.197
269	38	14.07	0	12	12	0.65	1.18	2.49	0.1689
270	38	14.07	0	8	12	0.65	1.18	2.49	0.1103

**Table 3:** The study's input and output statistics.

Statistical Index	LL (%)	PI (%)	S (%)	FA (%)	M (mol/l)	A/B	Na/Al	Si/Al	UCS (MPa)
Minimum	37.70	14.07	0.00	0.00	4.00	0.45	0.24	1.49	0.00
Maximum	116.00	88.46	50.00	20.00	15.00	0.85	1.98	2.49	24.26
Mean	65.02	40.02	16.22	1.87	12.31	0.62	1.16	1.68	5.73
Standard Deviation	32.52	30.97	12.96	4.28	2.74	0.15	0.45	0.33	6.46
Kurtosis	− 1.36	− 1.37	0.27	6.12	2.43	− 1.07	− 0.66	0.89	− 0.35
Skewness	0.57	0.56	0.91	2.58	− 1.54	0.25	− 0.21	1.56	0.89

**Figure 1:**
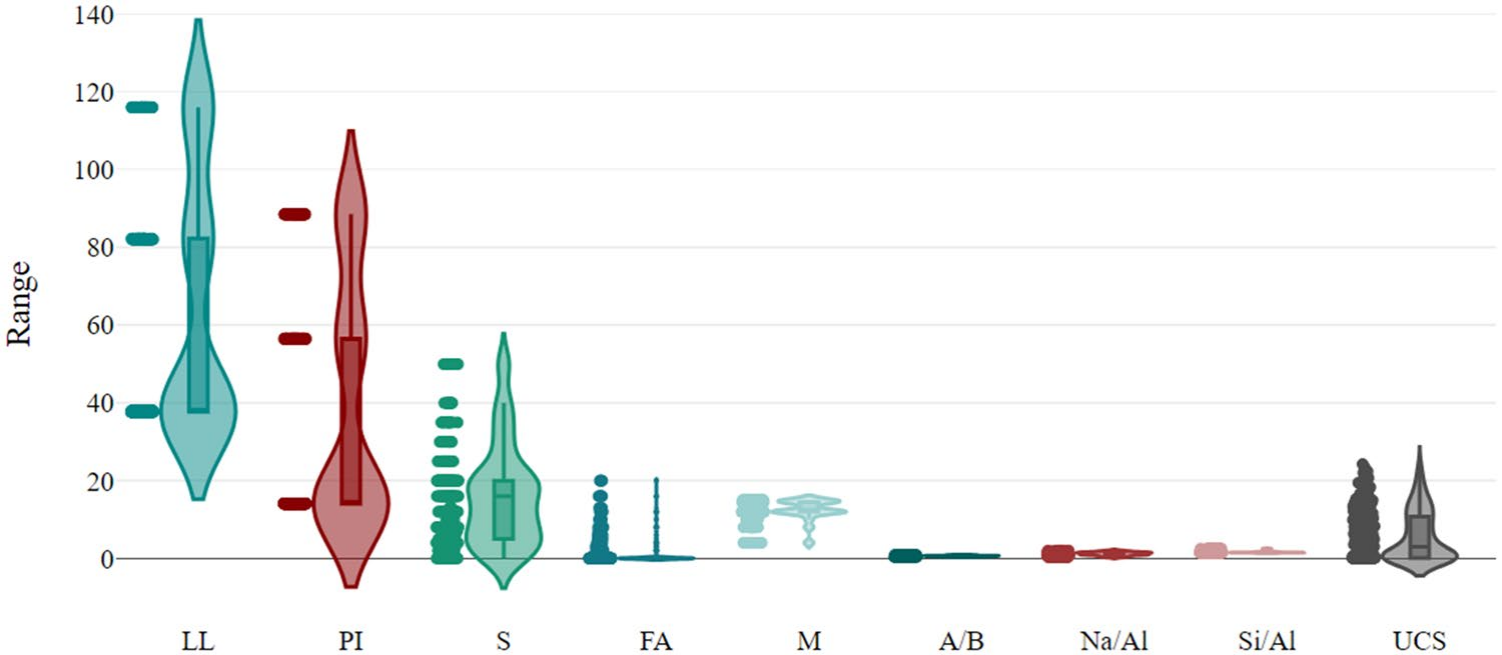
Violin plots of the dataset.

Figure 1 displays the violin plots of eight input variables and one output indicator. The visual representation consisted of a combination of box plots and sample points, serving to illustrate the comprehensive distribution of the dataset. In each violin plot, the upper and lower boundaries of the thick line correspond to the third and first quartiles of the samples, respectively. and, the upper and lower boundaries of the thin line reflect the upper and lower adjacent values. The findings depicted in Fig. 1 indicate that the distribution of S and UCS exhibited a rather balanced pattern, with their respective medians positioned towards the center of the violin plots. Conversely, LL, PI, FA, M, A/B, Na/Al, and Si/Al displayed a few individual outliers.

**Figure 2:**
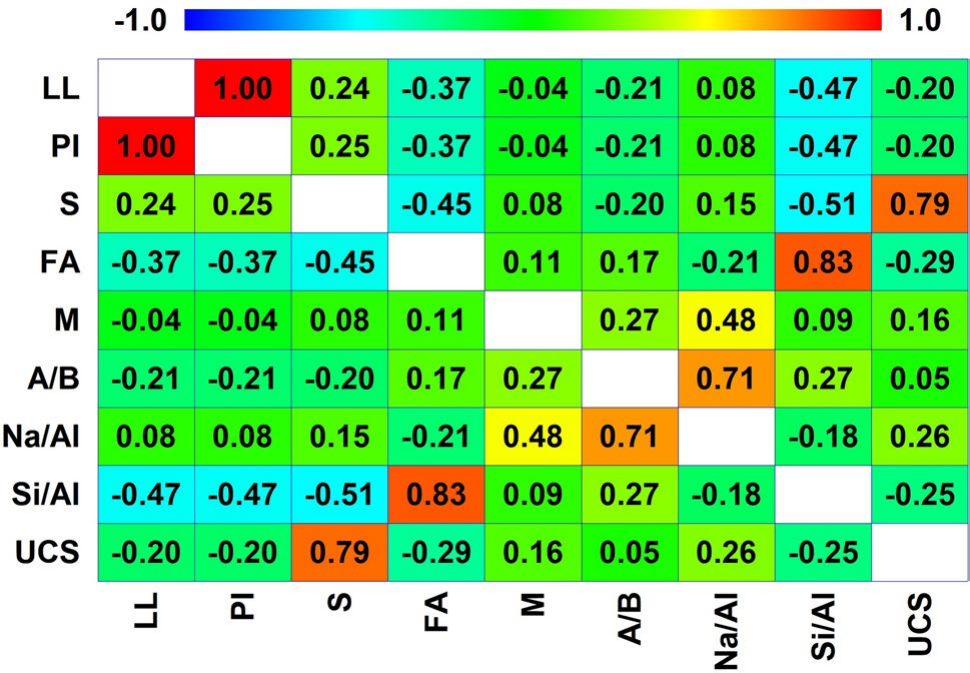
Correlation coefficients matrix diagram.

Correlation analysis analyzes the closeness of a relationship between two variables by analyzing two or more variables that are correlated. In order to ascertain the correlation among the input variables, this study performed a correlation analysis on the eight input variables prior to the training of the model. The resulting correlation analysis is presented in Fig. 2. The Pearson correlation coefficient showed that S has a strong positive correlation i.e., (*r* = 0.79) whereas exhibited a notably weak correlation (*r* = 0.05) with UCS of geopolymer stabilized clayey soil for the experimental data. The negative correlation (i.e., LL; *r* = − 0.20 and Si/Al; *r* = − 0.25) means a value increases with a decrease^49^.

## Machine learning models

Boosting is an ensemble approach in which several weak learners are combined to produce a strong learner. It reduces the errors of the previous learning model while improving the performance of the subsequent learning model. Gradient boosting (GB) and Adaptive Boosting (AdaBoost) are two often employed approaches among the several boosting methods available^42^. These two methods have been the focus of this study.

### Gradient boosting method

Gradient boosting (GB) is a type of ensemble method in which multiple weak models are developed and then combined to improve overall performance. Gradient Boosting (GB) uses the methodology of gradient descent in order to minimize the loss function that relates to a given model. The process of incorporating weak learners into the model is carried out using an iterative approach. The ultimate prediction is established by the combined input of every weak learner, which is then determined by a gradient optimization procedure with the objective of reducing the overall error of the strong learner^42,50^. Gradient boosting involves three fundamental mechanisms. The initial step that must be undertaken is the optimization of a loss function. A loss function that is differentiable is necessary. The degree of concordance between a machine learning model and observed data relevant to different phenomena is quantified using a loss function. The selection of the loss function may vary based on the particular problem under consideration. During the subsequent phase, the utilization of the weak learner is employed. The decision tree is utilized as the weak learner within gradient boosters. The application of regression trees that produce accurate values for divisions and allow for output aggregation is a distinctive approach used to handle residuals in prior iteration predictions by combining the output of consecutive models. While classification problems and regression concerns involve different methodologies, they both have a common approach in terms of data classification. The strategy employed for regression analysis involves the utilization of decision trees. The third phase entails the aggregation of numerous poor learners. Successive decision trees are incrementally incorporated into the analysis. The process of incorporating trees involves the implementation of a gradient descent technique in order to minimize loss. The gradient component is an essential part of gradient boosters. Instead of employing the parameters of the weaker models, the gradient descent optimization approach is utilized on the output of the model. The gradient boosting approach is an improved version of the gradient descent technique that enables generalization through the modification of both the gradient and the loss function^51^. The generic gradient boosting algorithm is represented in pseudocode as^52,53^:

**Figure:**
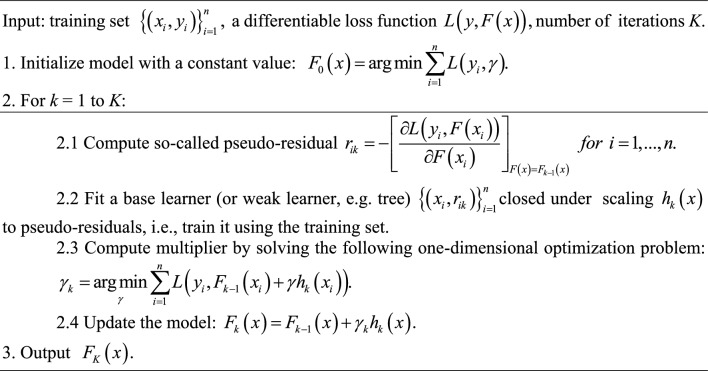
Algorithm 1: Gradient boosting.

### Adaptive boosting method

Adaptive boosting (AdaBoost), an ensemble of many weak learner decision trees, outperforms random guessing by a slight margin. The AdaBoost approach has an adaptive characteristic whereby it transfers the gradient information from prior trees to succeeding trees in order to minimize the error of the preceding tree. Consequently, the ongoing process of learning trees at each step fosters the development of a proficient student. The final prediction is determined by calculating the weighted average of the forecasts generated by each tree. In the process of training individual tree models, it is necessary to modify the weight distribution of each sample within the dataset. As the training data varies, the resulting training outcomes similarly exhibit variability, culminating in the aggregation of all outcomes^54^. AdaBoost demonstrates enhanced robustness against outliers and irrelevant data because to its notable adaptability. Furthermore, the methodology is specifically designed to operate in a manner where subsequent trees are provided with the information acquired by preceding trees. This enables them to focus exclusively on training samples that provide challenges in terms of prediction^55^.

Due to its constrained capabilities, a single decision tree is referred to as a weak learner. The possibility of generating a robust learner through the combination of numerous weaker learners is a subject of contemplation among researchers. The conjecture was proven in 1990, establishing the fundamental principles underlying the boosting algorithm, which involves the sequential combination of numerous weak learners^56^. The pseudocode of generic AdaBoost method is^57^:

**Figure:**
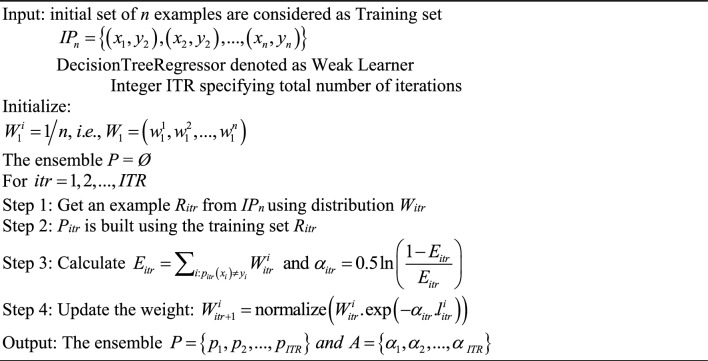
Algorithm 2: AdaBoost.

### Model evaluation

The selection of suitable evaluation metrics is of utmost importance in the development of different machine learning (ML) models, as it enables the assessment of the models' reliability and accuracy. The coefficient of determination (R^2^), mean absolute error (MAE), root mean square error (RMSE), and mean squared error (MSE) are commonly employed as evaluation metrics for regression models^58–61^. The coefficient of determination, denoted as R^2^, is a statistical metric that quantifies the proportion of the variance observed in the dependent variable that can be accounted for by the independent variables included in the model. The range of the metric is from 0 to 1, where higher values signify greater agreement between the model and the observed data. The MAE quantifies the average absolute discrepancy between the predicted values and the observed values, thereby serving as an indicator of the model's precision in predicting the target variable. On the other hand, the RMSE calculates the square root of the average squared discrepancy between the predicted values and the observed values. Lastly, the MSE computes the average squared discrepancy between the predicted and observed values. A decrease in the MAE, RMSE, and MSE indicates a higher level of model fit to the dataset. The evaluation measures chosen for the ML model in this work include R^2^, MAE, RMSE, and MSE. Table 4 provides definitions and calculation formulas. Furthermore, Taylor diagrams were employed to assess the efficacy of the models, thereby presenting both experimental and statistical parameters concurrently.

**Table 4:** Performance metrics definition and computation.

Performance metric	Definition	Formula
R^2^	The statistical metric that measures the proportion of the variance in the target variable	$R^{2} = 1 - \frac{{\sum\nolimits_{i = 1}^{m} {\left( {\hat{y}_{i} - y_{i} } \right)^{2} } }}{{\sum\nolimits_{i = 1}^{m} {\left( {\overline{y}_{i} - y_{i} } \right)^{2} } }}$
MAE	The average absolute difference between predicted and observed values	$MAE = \frac{1}{m}\sum\nolimits_{i = 1}^{m} {\left| {\hat{y}_{i} - y_{i} } \right|}$
RMSE	The root mean squared difference between predicted and measured values	$RMSE = \sqrt {\frac{1}{m}\sum\nolimits_{i = 1}^{m} {\left( {\hat{y}_{i} - y_{i} } \right)^{2} } }$
MSE	The mean squared difference between the observed and predicted values	$MSE = \frac{1}{m}\sum\nolimits_{i = 1}^{m} {\left( {\hat{y}_{i} - y_{i} } \right)^{2} }$

$\hat{y}_{i}$ represents the predicted value; $\overline{y}_{i}$ represents the average value; $y_{i}$ represents the measured value; and $m$ is the training or testing samples.

## Models development

**Figure 3:**
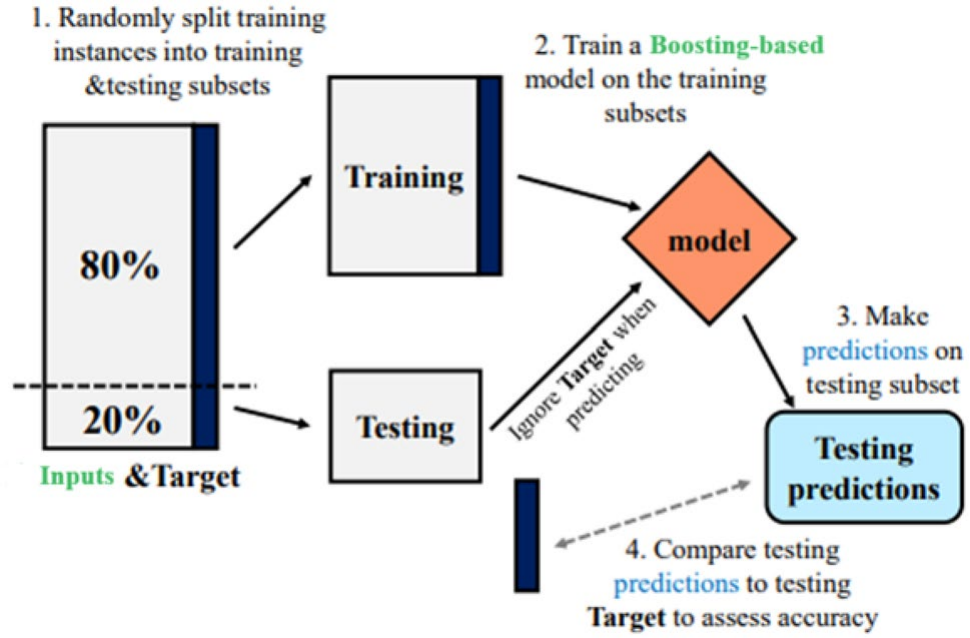
Flowchart of the proposed methodology (adopted from Wang et al.^63^).

Orange, a well-known open-source machine learning technology platform for statistical computation and data mining, was used for developing the models for predicting the UCS of geopolymer-stabilized clayey soil^62^. The data analysis in this study was conducted using Orange software (version 3.32.0), which was developed at the Bioinformatics Laboratory, Faculty of Computer and Information Science, University of Ljubljana, in collaboration with the open source community. Orange software incorporates a comprehensive range of ML algorithms that are widely utilized in research and practice. The database was randomly divided into training (80%; 216 samples) and testing (20%; 54 sample) sets for model development and validation. Random split aids in assessing data quality and addressing fairness concerns related to biased model predictions. The aforementioned methodologies were utilized in the building of our innovative ML models. The documentation provides a summary of the input parameters and implementation details for each ML method. It may be obtained at (https://orangedatamining.com/widget-catalog/, retrieved on 7 May 2023). The Orange platform offers a framework for the development of predictive modeling. Data normalization was not conducted. The schematic model, which was constructed using Orange Software, is depicted in Fig. 3. Additionally, Table 5 provides the specific values for each proposed model. The Orange 3 software used in this study does not have an optimizer function that can automatically determine the model's hyper-parameters. Consequently, the authors painstakingly fine-tuned the parameters of each ML model in Orange 3, beginning with the default values, in order to provide viable output. In the present study, the GB and AdaBoost models were fine-tuned by adjusting the important hyperparameters, as presented in Table 5. The initial selection of tuning parameter values for the models was followed by iterative adjustments during the trials, aiming to achieve the optimal fitness measures as presented in Table 5. Figure 3 depicts the schematic representation of the methods employed for the construction of the developed models.

**Table 5:** Selection of model parameters.

Model	Parameter	Explanation	Value
GB	Number of trees	How many gradient boosted trees will be included	100
	Learning rate	Boosting learning rate	0.1
	Limit depth of individual trees	Maximum depth of the individual tree	5.0
	Do not split subsets smaller than	Smallest subset that can be split	5.0
	Friction of training instances	Percentage of the training instances for fitting the individual tree	1.0
AdaBoost	Number of estimators	Maximum number of iterations of the weak learner	100
	Learning rate	The iteration pace may be slowed down if the step size of the update parameter is too small	1.0
	Classification algorithm	SAMME and SAMME.R classification algorithms update the weights of the base estimator with classification/probability outcomes	SAMME
	Regression loss function	There are three choices—linear, square and exponential	Linear

## Results and discussion

### Performance analysis

In the field of ML, it is necessary to examine models in order to verify the effectiveness of the obtained models. Different models utilize different evaluation approaches. After the successful development of the ML model for predicting the UCS of geopolymer stabilized clayey soil, the subsequent critical step entails evaluating the effectiveness of the generated ML model in producing accurate predictions. The primary objective of this work was to assess the validity of boosting-based models in accurately predicting the UCS of geopolymer stabilized clayey soil. The accomplishment was attained by a process of comparing the predicted values determined by the models with the measured or observed values of UCS.

**Figure 4:**
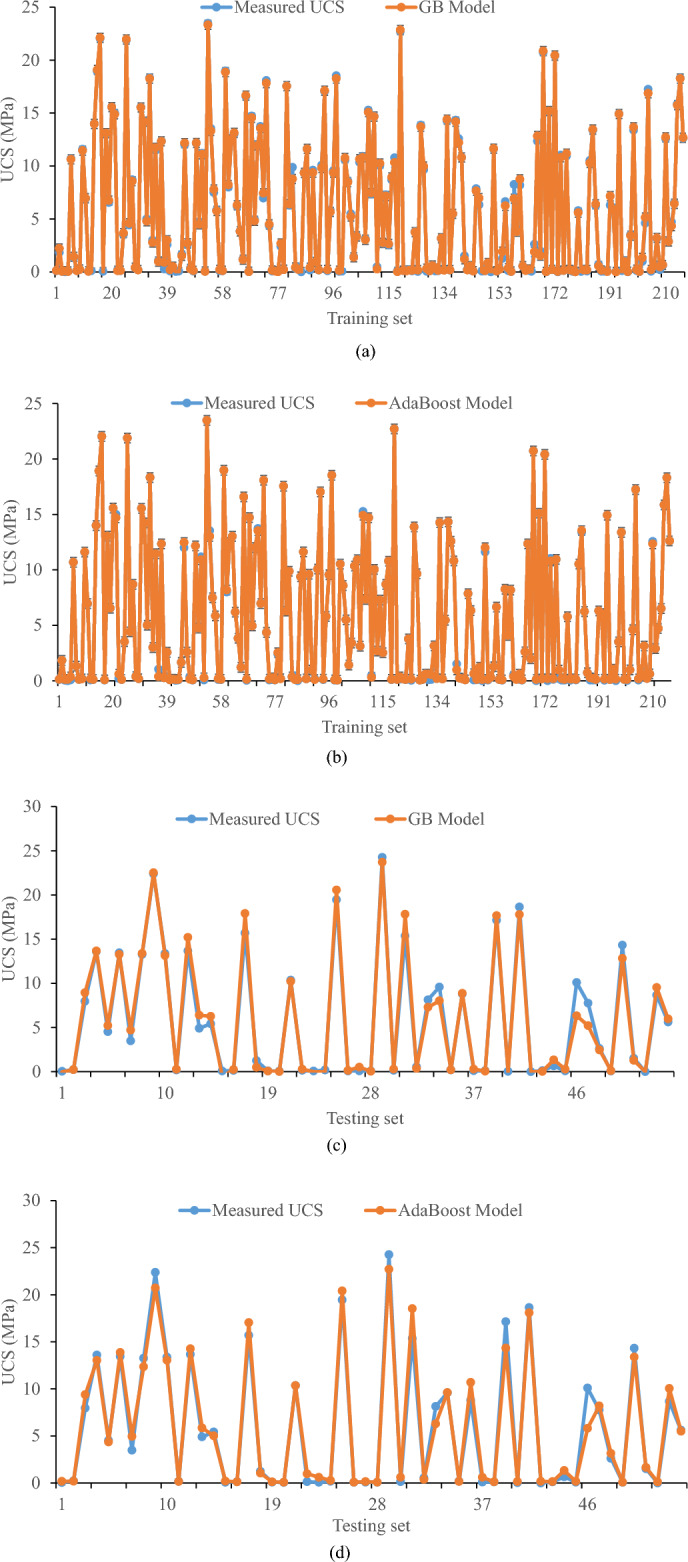
Comparing predicted and actual UCS of geopolymer stabilized clayey soil in training dataset using (**a**) GB, (**b**) AdaBoost and testing dataset using (**c**) GB and (**d**) AdaBoost.

Figure 4 illustrates a comparison between the predicted and measured unconfined compressive strength (UCS) of clayey soil stabilized with geopolymer. The comparison is made between the training and testing datasets. Figure 4 illustrates the excellent agreement observed between the predicted value of the training set and the actual measured value. Although there are some instances in the testing set when the predicted value exhibits substantial deviation from the measured value, on the whole, the predicted value coincides with the actual value. The results suggest that the GB model exhibits greater accuracy in predicting the UCS of geopolymer stabilized clayey soil when compared to the AdaBoost model.

**Figure 5:**
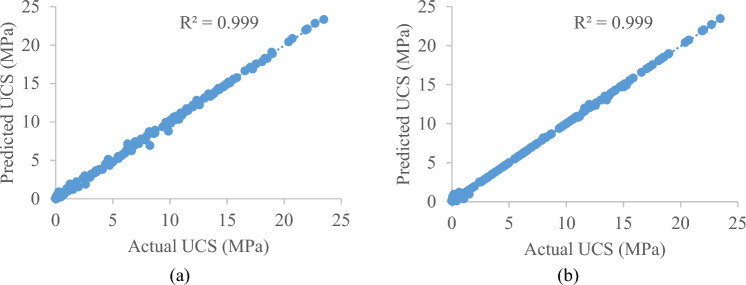
Actual/measured UCS of geopolymer stabilized clayey soil versus predicted UCS based on training dataset (**a**) GB model and (**b**) AdaBoost model.

**Figure 6:**
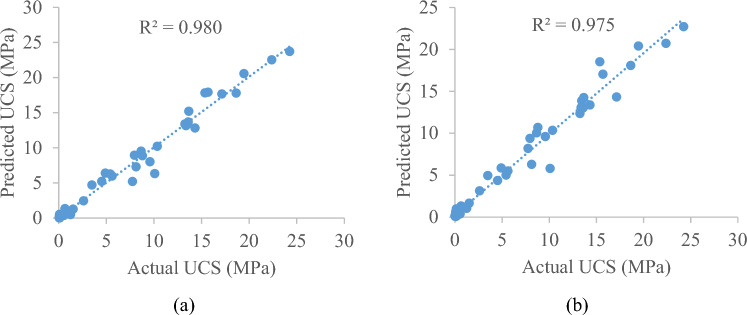
Measured/actual UCS of geopolymer stabilized clayey soil vs predicted UCS based on testing dataset (**a**) GB model and (**b**) AdaBoost model.

Figures 5 and 6 illustrate scatter diagrams that represent the fitting effect between predicted and measured values in both the training and testing sets, so providing an improved understanding of this relationship. The unconfined compressive strength of both the training set and the testing set is concentrated at 0–25 MPa, as can be seen in Figs. 5 and 6. The predicted value and measured value of the training set and the testing set, on the whole, have a good fitting effect, with only a few noise/error points in the testing set having significant errors. In the training set for GB model, there were multiple instances with errors such as the actual value of unconfined compressive strength was about 8.2621 MPa, and the predicted value was as low as 6.92243 MPa.

Nevertheless, it is important to note that slight variations in specific data points do not impact the overall predictive efficiency of the developed models. Specifically, the GB model demonstrates superior accuracy in predicting the UCS of geopolymer stabilized clayey soil compared to the AdaBoost model. The R^2^ value of the testing set is 0.980, the MAE value is 0.586, the RMSE value is 0.969, the MSE value is 0.940, whereas in AdaBoost model, The R^2^ value of the testing set is 0.975, the MAE value is 0.655, the RMSE value is 1.088, and the MSE value is 1.185. Thus, the R^2^ value, RMSE value, MAE value and MSE value of the testing set have common characteristics-namely, their R^2^ value is high, and their RMSE, MAE and MSE values are low in GB model as compared to AdaBoost model. In order to mitigate the risks of overfitting and ensuring that developed models remain robust and reliable, early stopping strategy is used i.e., stop training when the model's performance starts to degrade. It is evident from the results that the GB model has capable to predict the UCS accurately, and there is no over-fitting situation.

### Comparison between developed models with previously developed models

**Figure 7:**
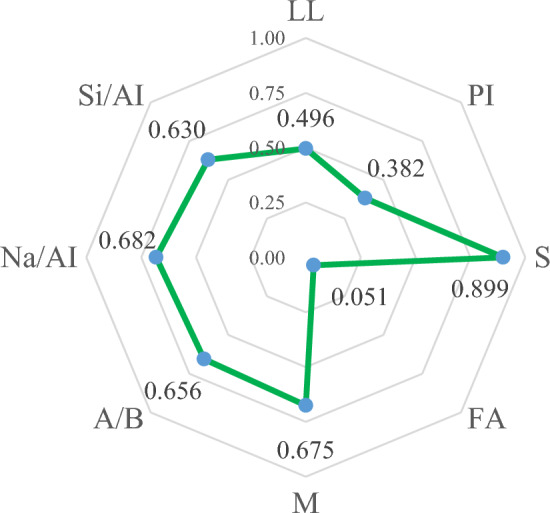
The strength relationship between effective parameters on UCS of geopolymer stabilized clayey soil.

**Figure 8:**
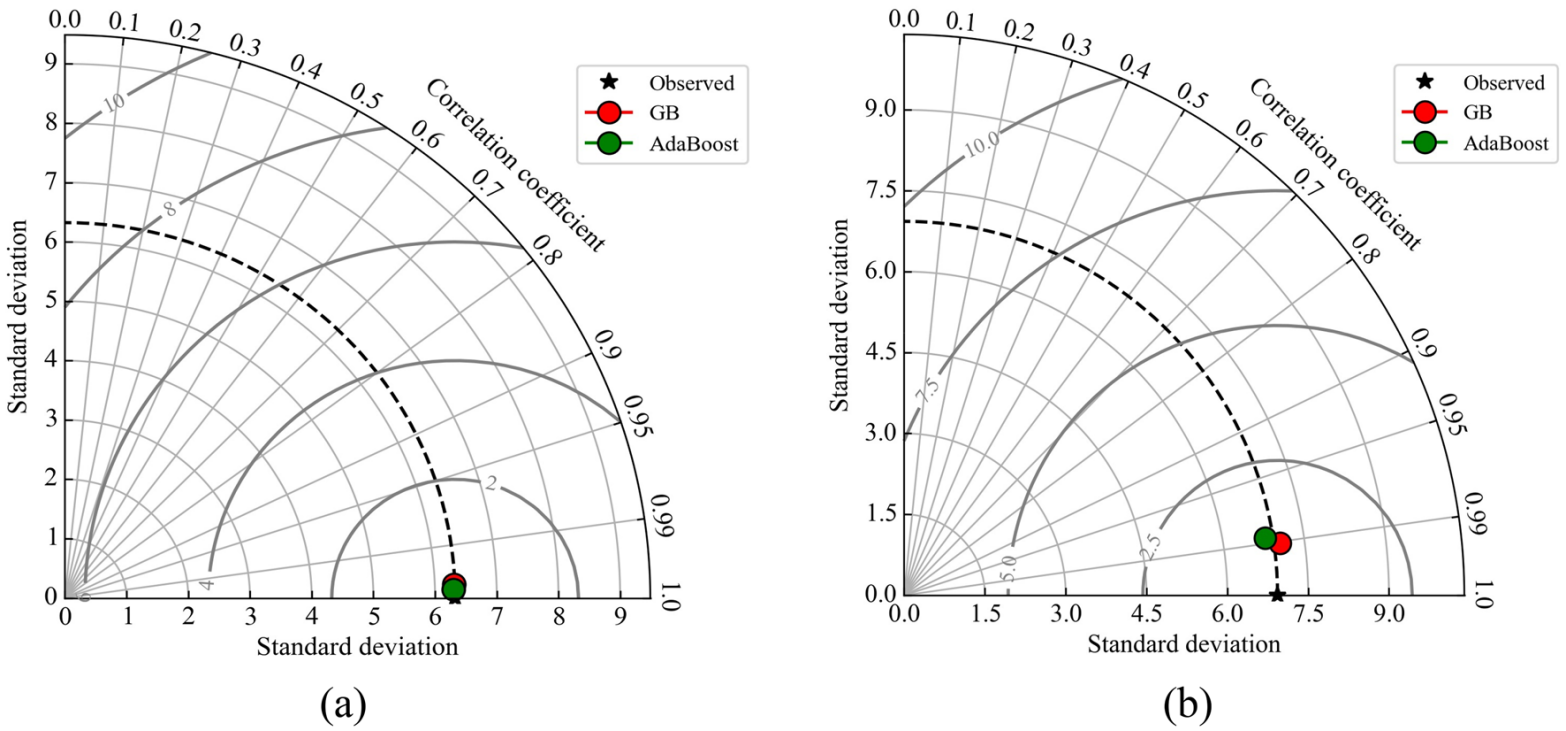
Taylor diagram comparing predicted and measured UCS of geopolymer stabilized clayey soil in (**a**) training and (**b**) testing datasets.

To highlight the predictive power of the developed models (i.e., GB and AdaBoost), the model results are compared to those of the most recently developed ML models in the literature in this study. A total of five machine learning models were taken into consideration: multivariable regression model (MLSR)^8^, MGGP^8^, RF^51^, extreme gradient boosting (XGB)^51^, and ANN^51^. Table 6 presents a comprehensive overview of the statistical performance metrics pertaining to both the pre-existing models and the newly developed models, specifically the GB and AdaBoost models, in terms of their predictive performance on the training and testing datasets. Figures 7 and 8 depict a scatter plot showing the relationship between the actual and predicted values of the UCS of geopolymer stabilized clayey soil. It is evident from the figures that all the developed models exhibit a satisfactory level of accuracy in predicting the UCS values of geopolymer stabilized clayey soil. Based on Table 6, the GB and AdaBoost models are efficient in predicting UCS values better than MLSR^34^, MGGP^34^, RF^39^, XGB^39^, and ANN^39^ techniques. However, the GB model with an R^2^ of 0.980 for the testing part was more accurate than the MGGP with an R^2^ of 0.922, MLSR with an R^2^ of 0.803, RF with an R^2^ of 0.9459, XGB with an R^2^ of 0.9671, ANN with an R^2^ of 0.9676 and AdaBoost with an R^2^ of 0.975. It can be concluded that the GB model is a superior model in predicting UCS values of geopolymer stabilized clayey soil. The GB model is found to be superior in predicting UCS values of geopolymer stabilized clayey soil. Furthermore, the GB model demonstrates the lowest MAE, RMSE, and MSE in the present investigation, indicating its superior efficiency and robustness.

**Table 6:** Comparison of the developed models with available machine learning models in literature.

Model	Dataset	R^2^	MAE (MPa)	RMSE (MPa)	MSE	Input Parameters	References
MGGP	Training	0.924	1.354	1.790	3.206	LL, PI, S, FA, M, A/B Na/Al, Si/Al	^8^
	Testing	0.922	1.305	–	3.149		
MLSR	Training	0.788	2.769	3.739	14.722		
	Testing	0.803	2.258	–	10.951		
RF	Training	0.9824	0.6051	0.8665	–	LL, PI, S, FA, M, A/B Na/Al, Si/Al	^51^
	Testing	0.9459	0.9706	1.4795	–		
XGB	Training	0.9929	0.3767	0.5500	–		
	Testing	0.9671	0.7357	1.1537	–		
ANN	Training	0.9883	0.4311	0.7084	–		
	Testing	0.9676	0.7140	1.1445	–		
GB	Training	0.999	0.145	0.229	0.052	LL, PI, S, FA, M, A/B Na/Al, Si/Al	This study
	Testing	0.980	0.586	0.969	0.940		
AdaBoost	Training	0.999	0.062	0.148	0.022		
	Testing	0.975	0.655	1.088	1.185		

“–” not reported in the respective reference.

### Sensitivity analysis

In the final stage of this study, the most and least significant parameters for determining the UCS of geopolymer-stabilized clayey soil were determined. Sensitivity analysis can help to understand which input features or variables have the most significant impact on the model's predictions. This can provide insights into which factors are driving the predictions, making the model more interpretable. This knowledge is vital for feature selection or understanding the underlying dynamics of the problem. In this study, cosine amplitude (CA) method of sensitivity analysis was used^64^. The CA approach has been employed in numerous research studies e.g.^65–68^.The CA approach measures the strength of the relationship between each effective parameter and the UCS of geopolymer stabilized clayey soil. The following equation is used in this context^69^.

1 $$ r_{ij} = \frac{{\sum\limits_{n = 1}^{m} {x_{in} \cdot x_{jn} } }}{{\sqrt {\left( {\sum\limits_{n = 1}^{m} {x_{in}^{2} } } \right) \cdot \left( {\sum\limits_{n = 1}^{m} {x_{jn}^{2} } } \right)} }} $$

In which *r*_*ij*_ is the intensity impact between *x*_*i*_ (input) and *x*_*j*_ (output). The sensitivity results and impactful parameters were determined. From Fig. 7, the S parameter has the most effect on unconfined compressive strength of geopolymer stabilized clayey soil with a strength of 0.899 and align with the finding reported from a previous study carried by Soleimani et al.^8^. Noteworthy, the influence of the parameters based on the *r*_*ij*_ value can be prioritized in ascending order as FA < PI < LL < Si/Al < A/B < M < Na/Al < S with an impact of 0.051, 0.382, 0.496, 0.630, 0.656, 0.675, 0.682, and 0.899, respectively.

### Taylor diagrams

Taylor diagram^70^ provides a straightforward visual representation of a model's performance in comparison to other models. The Taylor diagram incorporates three indices: the correlation coefficient, the standard deviation, and the root mean square error (RMSE). The Taylor diagram presented in Fig. 8 is utilized to conduct a comprehensive analysis of the model outcomes and facilitate a comparison between them. The Taylor diagram is a useful tool for visually representing the accuracy of prediction models. It accomplishes this by comparing various metrics such as the standard deviation, correlation coefficient, and RMSE. The evaluation of the performance of the created prediction models is also assessed using the Taylor diagram depicted in Fig. 8. The best model using a Taylor diagram based on standard deviation is the model that closely matches observed data in terms of standard deviation and have points closer to the reference point. AdaBoost's predictions are the closest to the observed/measured values in the training set. However, in case of testing dataset, it was seen that the GB model had somewhat superior performance compared to the AdaBoost model, suggesting that the GB model demonstrates a higher level of accuracy.

## Conclusions

This study examines the performance of boosting-based ML models in predicting the UCS of geopolymer stabilized clayey soil using experimental dataset. The dataset comprises input variables including fly ash and ground granulated blast furnace slag, liquid limit, plastic limit, plasticity index, molar concentration, alkali to binder ratio, and ratios of sodium and silicon to aluminum. The accuracy of the developed models was validated by examining R^2^, MAE, RMSE, and MSE values, as well as the predicted and actual values of the training and testing sets. The output variable was the UCS of geopolymer stabilized clayey soil. The results indicate that the GB algorithm performs well. The findings are summarized as follows:

1. The GB model can be used to predict the unconfined compressive strength of geopolymer stabilized clayey soil and achieved better prediction results when compared to the AdaBoost model developed in present study and other models such as MLSR, MGGP, RF, XGB, and ANN developed in literature.
2. Results showed that the R^2^ values of the GB model in training set and the test set were 0.999 and 0.980, respectively, and the MAE, RSME, and MSE values were 0.145, 0.229, and 0.052 for training and 0.586, 0.969, and 0.940 for testing set, respectively—that is, the training set and the testing set both had high R^2^ values and low MAE, RSME, and MSE values.
3. Based on the scatter plots of actual and predicted values, the GB model exhibited a better fit to the actual data, indicating that it has potential for broader applications in in predicting the UCS of geopolymer stabilized clayey soil.
4. The Pearson correlation coefficient showed that S and the UCS of geopolymer stabilized clayey soil have a strong positive correlation, while M and A/B have a very weak positive correlation.
5. The sensitivity analysis revealed that S held the highest level of significance in its contribution to UCS of geopolymer stabilized clayey soil. Moreover, Na/Al and M were identified as the subsequent key factors. In contrast, FA and PI demonstrated the least significance in the prediction of UCS values. The degree of importance can be prioritized in ascending order as FA < PI < LL < Si/Al < A/B < M < Na/Al < S.

The developed models are valid only within the considered ranges of inputs and should be verified beyond these ranges. In addition, more experimental data should be collected to improve the generalization capability of the proposed models. The prediction of unconfined compressive strength value of geopolymer stabilized clayey soil using sophisticated ML algorithms such as deep learning is left as a topic for future study.

### Supplementary Information

Supplementary Information.

## Data Availability

Data is provided within the manuscript or supplementary information files.

## Abbreviations

ANN: Artificial neural network
LL: Liquid limit
PL: Plastic limit
PI: Plasticity index
S: Ground granular blast furnace slag
FA: Fly ash
M: Molar concentration
A/B: Alkali to binder ratio
Na/Al: Ratio of Na to Al
Si/Al: Ratio of Si to Al
MAE: Mean absolute error
MSE: Mean squared error
ML: Machine learning
MGGP: Multi-gen genetic programming
MLSR: Multivariable regression model
MVR: Multivariable regression
RMSE: Root mean square error
SVM: Support vector machine
GB: Gradient boosting
AdaBoost: Adaptive boosting
UCS: Unconfined compressive strength
RF: Random forest
PSO: Particle swarm optimization
