# Supplementary material for: Boosting-based ensemble machine learning models for predicting unconfined compressive strength of geopolymer stabilized clayey soil
Source: Sci Rep. 2024 Jan 28;14:2323. doi: 10.1038/s41598-024-52825-7 (PMC10822860; doi:10.1038/s41598-024-52825-7)
Supplement: Supplementary file 1 — Supplementary Information. [file 41598_2024_52825_MOESM1_ESM.docx]

**Supplementary File**

**Appendix A**

**Table A1.** Experimental data used for modeling.

| **S. No.** | **LL (%)** | **PI (%)** | **S (%)** | **FA (%)** | **M (mol/l)** | **A/B** | **Na/Al** | **Si/Al** | **UCS (MPa)** |
| --- | --- | --- | --- | --- | --- | --- | --- | --- | --- |
| 1 | 116 | 88.46 | 20 | 0 | 4 | 0.45 | 0.39 | 1.49 | 0.0595 |
| 2 | 116 | 88.46 | 16 | 0 | 4 | 0.45 | 0.39 | 1.49 | 0.0616 |
| 3 | 116 | 88.46 | 12 | 0 | 4 | 0.45 | 0.39 | 1.49 | 0.0551 |
| 4 | 116 | 88.46 | 8 | 0 | 4 | 0.45 | 0.39 | 1.49 | 0.0494 |
| 5 | 116 | 88.46 | 4 | 0 | 4 | 0.45 | 0.39 | 1.49 | 0.0484 |
| 6 | 116 | 88.46 | 20 | 0 | 8 | 0.45 | 0.69 | 1.49 | 0.1784 |
| 7 | 116 | 88.46 | 16 | 0 | 8 | 0.45 | 0.69 | 1.49 | 0.1044 |
| 8 | 116 | 88.46 | 12 | 0 | 8 | 0.45 | 0.69 | 1.49 | 0.0542 |
| 9 | 116 | 88.46 | 8 | 0 | 8 | 0.45 | 0.69 | 1.49 | 0.0638 |
| 10 | 116 | 88.46 | 4 | 0 | 8 | 0.45 | 0.69 | 1.49 | 0.0553 |
| 11 | 116 | 88.46 | 20 | 0 | 12 | 0.45 | 0.93 | 1.49 | 1.836 |
| 12 | 116 | 88.46 | 16 | 0 | 12 | 0.45 | 0.93 | 1.49 | 1.5198 |
| 13 | 116 | 88.46 | 12 | 0 | 12 | 0.45 | 0.93 | 1.49 | 0.442 |
| 14 | 116 | 88.46 | 8 | 0 | 12 | 0.45 | 0.93 | 1.49 | 0.0738 |
| 15 | 116 | 88.46 | 4 | 0 | 12 | 0.45 | 0.93 | 1.49 | 0.0557 |
| 16 | 116 | 88.46 | 20 | 0 | 12 | 0.65 | 1.34 | 1.49 | 3.8335 |
| 17 | 116 | 88.46 | 16 | 0 | 12 | 0.65 | 1.34 | 1.49 | 2.5293 |
| 18 | 116 | 88.46 | 12 | 0 | 12 | 0.65 | 1.34 | 1.49 | 1.5138 |
| 19 | 116 | 88.46 | 8 | 0 | 12 | 0.65 | 1.34 | 1.49 | 0.4841 |
| 20 | 116 | 88.46 | 4 | 0 | 12 | 0.65 | 1.34 | 1.49 | 0 |
| 21 | 116 | 88.46 | 20 | 0 | 12 | 0.85 | 1.75 | 1.49 | 4.0932 |
| 22 | 116 | 88.46 | 16 | 0 | 12 | 0.85 | 1.75 | 1.49 | 3.0047 |
| 23 | 116 | 88.46 | 12 | 0 | 12 | 0.85 | 1.75 | 1.49 | 1.2701 |
| 24 | 116 | 88.46 | 8 | 0 | 12 | 0.85 | 1.75 | 1.49 | 0.5495 |
| 25 | 116 | 88.46 | 4 | 0 | 12 | 0.85 | 1.75 | 1.49 | 0.3633 |
| 26 | 116 | 88.46 | 20 | 0 | 15 | 0.45 | 1.05 | 1.49 | 3.7829 |
| 27 | 116 | 88.46 | 16 | 0 | 15 | 0.45 | 1.05 | 1.49 | 2.4024 |
| 28 | 116 | 88.46 | 12 | 0 | 15 | 0.45 | 1.05 | 1.49 | 1.2396 |
| 29 | 116 | 88.46 | 8 | 0 | 15 | 0.45 | 1.05 | 1.49 | 0.2017 |
| 30 | 116 | 88.46 | 4 | 0 | 15 | 0.45 | 1.05 | 1.49 | 0.064 |
| 31 | 116 | 88.46 | 20 | 0 | 15 | 0.65 | 1.52 | 1.49 | 4.7478 |
| 32 | 116 | 88.46 | 16 | 0 | 15 | 0.65 | 1.52 | 1.49 | 3.1467 |
| 33 | 116 | 88.46 | 12 | 0 | 15 | 0.65 | 1.52 | 1.49 | 1.6606 |
| 34 | 116 | 88.46 | 8 | 0 | 15 | 0.65 | 1.52 | 1.49 | 0.5452 |
| 35 | 116 | 88.46 | 4 | 0 | 15 | 0.65 | 1.52 | 1.49 | 0.0513 |
| 36 | 116 | 88.46 | 20 | 0 | 15 | 0.85 | 1.98 | 1.49 | 3.1376 |
| 37 | 116 | 88.46 | 16 | 0 | 15 | 0.85 | 1.98 | 1.49 | 2.6131 |
| 38 | 116 | 88.46 | 12 | 0 | 15 | 0.85 | 1.98 | 1.49 | 1.4259 |
| 39 | 116 | 88.46 | 8 | 0 | 15 | 0.85 | 1.98 | 1.49 | 0.635 |
| 40 | 116 | 88.46 | 4 | 0 | 15 | 0.85 | 1.98 | 1.49 | 0.0656 |
| 41 | 82 | 56.46 | 20 | 0 | 4 | 0.45 | 0.39 | 1.49 | 0.0575 |
| 42 | 82 | 56.46 | 16 | 0 | 4 | 0.45 | 0.39 | 1.49 | 0.0568 |
| 43 | 82 | 56.46 | 12 | 0 | 4 | 0.45 | 0.39 | 1.49 | 0.0617 |
| 44 | 82 | 56.46 | 8 | 0 | 4 | 0.45 | 0.39 | 1.49 | 0.0724 |
| 45 | 37.7 | 14.07 | 0 | 12 | 14.5 | 0.65 | 1.34 | 2.49 | 0.18 |
| 46 | 37.7 | 14.07 | 0 | 8 | 14.5 | 0.65 | 1.34 | 2.49 | 0.127 |
| 47 | 37.7 | 14.07 | 0 | 4 | 14.5 | 0.65 | 1.34 | 2.49 | 0 |
| 48 | 37.7 | 14.07 | 0 | 20 | 14.5 | 0.85 | 1.75 | 2.49 | 0.189 |
| 49 | 37.7 | 14.07 | 0 | 16 | 14.5 | 0.85 | 1.75 | 2.49 | 0.17 |
| 50 | 37.7 | 14.07 | 0 | 12 | 14.5 | 0.85 | 1.75 | 2.49 | 0.163 |
| 51 | 37.7 | 14.07 | 0 | 8 | 14.5 | 0.85 | 1.75 | 2.49 | 0.12 |
| 52 | 37.7 | 14.07 | 0 | 4 | 14.5 | 0.85 | 1.75 | 2.49 | 0 |
| 53 | 37.7 | 14.07 | 8 | 12 | 12 | 0.65 | 0.49 | 2.12 | 5.855 |
| 54 | 37.7 | 14.07 | 6 | 10 | 12 | 0.65 | 0.49 | 2.12 | 2.597 |
| 55 | 37.7 | 14.07 | 5 | 7 | 12 | 0.65 | 0.49 | 2.12 | 0.12 |
| 56 | 37.7 | 14.07 | 3 | 5 | 12 | 0.65 | 0.49 | 2.12 | 0.097 |
| 57 | 37.7 | 14.07 | 2 | 2 | 12 | 0.65 | 0.49 | 2.12 | 0.053 |
| 58 | 37.7 | 14.07 | 8 | 12 | 14.5 | 0.65 | 0.56 | 2.12 | 4.899 |
| 59 | 37.7 | 14.07 | 6 | 10 | 14.5 | 0.65 | 0.56 | 2.12 | 3.41 |
| 60 | 37.7 | 14.07 | 5 | 7 | 14.5 | 0.65 | 0.56 | 2.12 | 0.604 |
| 61 | 37.7 | 14.07 | 3 | 5 | 14.5 | 0.65 | 0.56 | 2.12 | 0.096 |
| 62 | 37.7 | 14.07 | 2 | 2 | 14.5 | 0.65 | 0.56 | 2.12 | 0.074 |
| 63 | 37.7 | 14.07 | 12 | 8 | 12 | 0.65 | 0.76 | 1.92 | 7.346 |
| 64 | 37.7 | 14.07 | 10 | 6 | 12 | 0.65 | 0.76 | 1.92 | 6.191 |
| 65 | 37.7 | 14.07 | 7 | 5 | 12 | 0.65 | 0.76 | 1.92 | 0.348 |
| 66 | 37.7 | 14.07 | 5 | 3 | 12 | 0.65 | 0.76 | 1.92 | 0.144 |
| 67 | 37.7 | 14.07 | 2 | 2 | 12 | 0.65 | 0.76 | 1.92 | 0.045 |
| 68 | 37.7 | 14.07 | 12 | 8 | 14.5 | 0.65 | 0.86 | 1.92 | 9.562 |
| 69 | 37.7 | 14.07 | 10 | 6 | 14.5 | 0.65 | 0.86 | 1.92 | 8.595 |
| 70 | 37.7 | 14.07 | 7 | 5 | 14.5 | 0.65 | 0.86 | 1.92 | 0.971 |
| 71 | 37.7 | 14.07 | 5 | 3 | 14.5 | 0.65 | 0.86 | 1.92 | 0.14 |
| 72 | 37.7 | 14.07 | 2 | 2 | 14.5 | 0.65 | 0.86 | 1.92 | 0.054 |
| 73 | 37.7 | 14.07 | 4 | 16 | 12 | 0.65 | 0.24 | 2.31 | 2.687 |
| 74 | 37.7 | 14.07 | 3 | 13 | 12 | 0.65 | 0.24 | 2.31 | 0.65 |
| 75 | 37.7 | 14.07 | 2 | 10 | 12 | 0.65 | 0.24 | 2.31 | 0.199 |
| 76 | 37.7 | 14.07 | 2 | 6 | 12 | 0.65 | 0.24 | 2.31 | 0.109 |
| 77 | 37.7 | 14.07 | 1 | 3 | 12 | 0.65 | 0.24 | 2.31 | 0.037 |
| 78 | 37.7 | 14.07 | 4 | 16 | 14.5 | 0.65 | 0.27 | 2.31 | 3.127 |
| 79 | 37.7 | 14.07 | 3 | 13 | 14.5 | 0.65 | 0.27 | 2.31 | 0.615 |
| 80 | 37.7 | 14.07 | 2 | 10 | 14.5 | 0.65 | 0.27 | 2.31 | 0.134 |
| 81 | 37.7 | 14.07 | 2 | 6 | 14.5 | 0.65 | 0.27 | 2.31 | 0.142 |
| 82 | 37.7 | 14.07 | 1 | 3 | 14.5 | 0.65 | 0.27 | 2.31 | 0 |
| 83 | 37.7 | 14.07 | 16 | 4 | 12 | 0.65 | 1.04 | 1.7 | 10.53 |
| 84 | 37.7 | 14.07 | 13 | 3 | 12 | 0.65 | 1.04 | 1.7 | 7.492 |
| 85 | 37.7 | 14.07 | 10 | 2 | 12 | 0.65 | 1.04 | 1.7 | 0.656 |
| 86 | 37.7 | 14.07 | 6 | 2 | 12 | 0.65 | 1.04 | 1.7 | 0.24 |
| 87 | 37.7 | 14.07 | 3 | 1 | 12 | 0.65 | 1.04 | 1.7 | 0 |
| 88 | 37.7 | 14.07 | 16 | 4 | 14.5 | 0.65 | 1.18 | 1.7 | 10.56 |
| 89 | 82 | 56.46 | 4 | 0 | 4 | 0.45 | 0.39 | 1.49 | 0.0761 |
| 90 | 82 | 56.46 | 20 | 0 | 8 | 0.45 | 0.69 | 1.49 | 2.6153 |
| 91 | 82 | 56.46 | 16 | 0 | 8 | 0.45 | 0.69 | 1.49 | 0.7228 |
| 92 | 82 | 56.46 | 12 | 0 | 8 | 0.45 | 0.69 | 1.49 | 0.081 |
| 93 | 82 | 56.46 | 8 | 0 | 8 | 0.45 | 0.69 | 1.49 | 0.0815 |
| 94 | 82 | 56.46 | 4 | 0 | 8 | 0.45 | 0.69 | 1.49 | 0.0611 |
| 95 | 82 | 56.46 | 20 | 0 | 12 | 0.45 | 0.93 | 1.49 | 4.7157 |
| 96 | 82 | 56.46 | 16 | 0 | 12 | 0.45 | 0.93 | 1.49 | 0.9838 |
| 97 | 82 | 56.46 | 12 | 0 | 12 | 0.45 | 0.93 | 1.49 | 0.1699 |
| 98 | 82 | 56.46 | 8 | 0 | 12 | 0.45 | 0.93 | 1.49 | 0.092 |
| 99 | 82 | 56.46 | 4 | 0 | 12 | 0.45 | 0.93 | 1.49 | 0.1138 |
| 100 | 82 | 56.46 | 20 | 0 | 12 | 0.65 | 1.34 | 1.49 | 6.3339 |
| 101 | 82 | 56.46 | 16 | 0 | 12 | 0.65 | 1.34 | 1.49 | 5.499 |
| 102 | 82 | 56.46 | 12 | 0 | 12 | 0.65 | 1.34 | 1.49 | 2.9055 |
| 103 | 82 | 56.46 | 8 | 0 | 12 | 0.65 | 1.34 | 1.49 | 0.3208 |
| 104 | 82 | 56.46 | 4 | 0 | 12 | 0.65 | 1.34 | 1.49 | 0.1083 |
| 105 | 82 | 56.46 | 20 | 0 | 12 | 0.85 | 1.75 | 1.49 | 6.5492 |
| 106 | 82 | 56.46 | 16 | 0 | 12 | 0.85 | 1.75 | 1.49 | 5.612 |
| 107 | 82 | 56.46 | 12 | 0 | 12 | 0.85 | 1.75 | 1.49 | 4.5196 |
| 108 | 82 | 56.46 | 8 | 0 | 12 | 0.85 | 1.75 | 1.49 | 1.0488 |
| 109 | 82 | 56.46 | 4 | 0 | 12 | 0.85 | 1.75 | 1.49 | 0.0826 |
| 110 | 82 | 56.46 | 20 | 0 | 15 | 0.45 | 1.05 | 1.49 | 6.9624 |
| 111 | 82 | 56.46 | 16 | 0 | 15 | 0.45 | 1.05 | 1.49 | 4.4171 |
| 112 | 82 | 56.46 | 12 | 0 | 15 | 0.45 | 1.05 | 1.49 | 1.0497 |
| 113 | 82 | 56.46 | 8 | 0 | 15 | 0.45 | 1.05 | 1.49 | 0.1081 |
| 114 | 82 | 56.46 | 4 | 0 | 15 | 0.45 | 1.05 | 1.49 | 0.0969 |
| 115 | 82 | 56.46 | 20 | 0 | 15 | 0.65 | 1.52 | 1.49 | 7.8573 |
| 116 | 82 | 56.46 | 16 | 0 | 15 | 0.65 | 1.52 | 1.49 | 6.0593 |
| 117 | 82 | 56.46 | 12 | 0 | 15 | 0.65 | 1.52 | 1.49 | 3.5162 |
| 118 | 82 | 56.46 | 8 | 0 | 15 | 0.65 | 1.52 | 1.49 | 0.3799 |
| 119 | 82 | 56.46 | 4 | 0 | 15 | 0.65 | 1.52 | 1.49 | 0.1194 |
| 120 | 82 | 56.46 | 20 | 0 | 15 | 0.85 | 1.98 | 1.49 | 6.9127 |
| 121 | 82 | 56.46 | 16 | 0 | 15 | 0.85 | 1.98 | 1.49 | 5.7791 |
| 122 | 82 | 56.46 | 12 | 0 | 15 | 0.85 | 1.98 | 1.49 | 4.3611 |
| 123 | 82 | 56.46 | 8 | 0 | 15 | 0.85 | 1.98 | 1.49 | 1.9891 |
| 124 | 82 | 56.46 | 4 | 0 | 15 | 0.85 | 1.98 | 1.49 | 0.0786 |
| 125 | 38 | 14.07 | 20 | 0 | 4 | 0.45 | 0.39 | 1.49 | 0.0724 |
| 126 | 38 | 14.07 | 16 | 0 | 4 | 0.45 | 0.39 | 1.49 | 0.0778 |
| 127 | 38 | 14.07 | 12 | 0 | 4 | 0.45 | 0.39 | 1.49 | 0.0713 |
| 128 | 38 | 14.07 | 8 | 0 | 4 | 0.45 | 0.39 | 1.49 | 0.0445 |
| 129 | 38 | 14.07 | 4 | 0 | 4 | 0.45 | 0.39 | 1.49 | 0.0301 |
| 130 | 38 | 14.07 | 20 | 0 | 8 | 0.45 | 0.69 | 1.49 | 0.2017 |
| 131 | 38 | 14.07 | 16 | 0 | 8 | 0.45 | 0.69 | 1.49 | 0.1687 |
| 132 | 38 | 14.07 | 12 | 0 | 8 | 0.45 | 0.69 | 1.49 | 0.1089 |
| 133 | 38 | 14.07 | 8 | 0 | 8 | 0.45 | 0.69 | 1.49 | 0.0869 |
| 134 | 37.7 | 14.07 | 13 | 3 | 14.5 | 0.65 | 1.18 | 1.7 | 9.61 |
| 135 | 37.7 | 14.07 | 10 | 2 | 14.5 | 0.65 | 1.18 | 1.7 | 1.323 |
| 136 | 37.7 | 14.07 | 6 | 2 | 14.5 | 0.65 | 1.18 | 1.7 | 0.176 |
| 137 | 37.7 | 14.07 | 3 | 1 | 14.5 | 0.65 | 1.18 | 1.7 | 0.04 |
| 138 | 116 | 88.46 | 25 | 0 | 12 | 0.45 | 0.93 | 1.49 | 5.012 |
| 139 | 116 | 88.46 | 30 | 0 | 12 | 0.45 | 0.93 | 1.49 | 6.644 |
| 140 | 116 | 88.46 | 35 | 0 | 12 | 0.45 | 0.93 | 1.49 | 7.278 |
| 141 | 116 | 88.46 | 40 | 0 | 12 | 0.45 | 0.93 | 1.49 | 8.696 |
| 142 | 116 | 88.46 | 50 | 0 | 12 | 0.45 | 0.93 | 1.49 | 10.35 |
| 143 | 116 | 88.46 | 25 | 0 | 12 | 0.65 | 1.34 | 1.49 | 5.423 |
| 144 | 116 | 88.46 | 30 | 0 | 12 | 0.65 | 1.34 | 1.49 | 6.251 |
| 145 | 116 | 88.46 | 35 | 0 | 12 | 0.65 | 1.34 | 1.49 | 8.13 |
| 146 | 116 | 88.46 | 40 | 0 | 12 | 0.65 | 1.34 | 1.49 | 9.384 |
| 147 | 116 | 88.46 | 50 | 0 | 12 | 0.65 | 1.34 | 1.49 | 11.18 |
| 148 | 116 | 88.46 | 25 | 0 | 14.5 | 0.45 | 1.05 | 1.49 | 3.484 |
| 149 | 116 | 88.46 | 30 | 0 | 14.5 | 0.45 | 1.05 | 1.49 | 4.605 |
| 150 | 116 | 88.46 | 35 | 0 | 14.5 | 0.45 | 1.05 | 1.49 | 6.519 |
| 151 | 116 | 88.46 | 40 | 0 | 14.5 | 0.45 | 1.05 | 1.49 | 8.7 |
| 152 | 116 | 88.46 | 50 | 0 | 14.5 | 0.45 | 1.05 | 1.49 | 10.35 |
| 153 | 116 | 88.46 | 25 | 0 | 14.5 | 0.65 | 1.52 | 1.49 | 4.951 |
| 154 | 116 | 88.46 | 30 | 0 | 14.5 | 0.65 | 1.52 | 1.49 | 5.471 |
| 155 | 116 | 88.46 | 35 | 0 | 14.5 | 0.65 | 1.52 | 1.49 | 6.285 |
| 156 | 116 | 88.46 | 40 | 0 | 14.5 | 0.65 | 1.52 | 1.49 | 7.956 |
| 157 | 116 | 88.46 | 50 | 0 | 14.5 | 0.65 | 1.52 | 1.49 | 10.92 |
| 158 | 82.2 | 56.46 | 25 | 0 | 12 | 0.45 | 0.93 | 1.49 | 6.304 |
| 159 | 82.2 | 56.46 | 30 | 0 | 12 | 0.45 | 0.93 | 1.49 | 11.62 |
| 160 | 82.2 | 56.46 | 35 | 0 | 12 | 0.45 | 0.93 | 1.49 | 13.87 |
| 161 | 82.2 | 56.46 | 40 | 0 | 12 | 0.45 | 0.93 | 1.49 | 15.55 |
| 162 | 82.2 | 56.46 | 50 | 0 | 12 | 0.45 | 0.93 | 1.49 | 18.53 |
| 163 | 82.2 | 56.46 | 25 | 0 | 12 | 0.65 | 1.34 | 1.49 | 10.69 |
| 164 | 82.2 | 56.46 | 30 | 0 | 12 | 0.65 | 1.34 | 1.49 | 12.35 |
| 165 | 82.2 | 56.46 | 35 | 0 | 12 | 0.65 | 1.34 | 1.49 | 13.26 |
| 166 | 82.2 | 56.46 | 40 | 0 | 12 | 0.65 | 1.34 | 1.49 | 13.66 |
| 167 | 82.2 | 56.46 | 50 | 0 | 12 | 0.65 | 1.34 | 1.49 | 15.69 |
| 168 | 82.2 | 56.46 | 25 | 0 | 14.5 | 0.45 | 1.05 | 1.49 | 10.08 |
| 169 | 82.2 | 56.46 | 30 | 0 | 14.5 | 0.45 | 1.05 | 1.49 | 12.59 |
| 170 | 82.2 | 56.46 | 35 | 0 | 14.5 | 0.45 | 1.05 | 1.49 | 13.45 |
| 171 | 82.2 | 56.46 | 40 | 0 | 14.5 | 0.45 | 1.05 | 1.49 | 15.12 |
| 172 | 82.2 | 56.46 | 50 | 0 | 14.5 | 0.45 | 1.05 | 1.49 | 15.36 |
| 173 | 82.2 | 56.46 | 25 | 0 | 14.5 | 0.65 | 1.52 | 1.49 | 8.791 |
| 174 | 82.2 | 56.46 | 30 | 0 | 14.5 | 0.65 | 1.52 | 1.49 | 10.95 |
| 175 | 82.2 | 56.46 | 35 | 0 | 14.5 | 0.65 | 1.52 | 1.49 | 12.02 |
| 176 | 82.2 | 56.46 | 40 | 0 | 14.5 | 0.65 | 1.52 | 1.49 | 14.26 |
| 177 | 82.2 | 56.46 | 50 | 0 | 14.5 | 0.65 | 1.52 | 1.49 | 17.04 |
| 178 | 37.7 | 14.07 | 25 | 0 | 12 | 0.45 | 0.93 | 1.49 | 15.86 |
| 179 | 38 | 14.07 | 4 | 0 | 8 | 0.45 | 0.69 | 1.49 | 0.0985 |
| 180 | 38 | 14.07 | 20 | 0 | 12 | 0.45 | 0.93 | 1.49 | 10.089 |
| 181 | 38 | 14.07 | 16 | 0 | 12 | 0.45 | 0.93 | 1.49 | 5.8037 |
| 182 | 38 | 14.07 | 12 | 0 | 12 | 0.45 | 0.93 | 1.49 | 0.1173 |
| 183 | 38 | 14.07 | 8 | 0 | 12 | 0.45 | 0.93 | 1.49 | 0.0676 |
| 184 | 38 | 14.07 | 4 | 0 | 12 | 0.45 | 0.93 | 1.49 | 0.1071 |
| 185 | 38 | 14.07 | 20 | 0 | 12 | 0.65 | 1.34 | 1.49 | 10.786 |
| 186 | 38 | 14.07 | 16 | 0 | 12 | 0.65 | 1.34 | 1.49 | 9.5833 |
| 187 | 38 | 14.07 | 12 | 0 | 12 | 0.65 | 1.34 | 1.49 | 2.5673 |
| 188 | 38 | 14.07 | 8 | 0 | 12 | 0.65 | 1.34 | 1.49 | 0.0693 |
| 189 | 38 | 14.07 | 4 | 0 | 12 | 0.65 | 1.34 | 1.49 | 0.0257 |
| 190 | 38 | 14.07 | 0 | 4 | 12 | 0.65 | 1.18 | 2.49 | 0 |
| 191 | 38 | 14.07 | 0 | 20 | 12 | 0.85 | 1.55 | 2.49 | 0.2293 |
| 192 | 38 | 14.07 | 0 | 16 | 12 | 0.85 | 1.55 | 2.49 | 0.2241 |
| 193 | 38 | 14.07 | 0 | 12 | 12 | 0.85 | 1.55 | 2.49 | 0.1868 |
| 194 | 38 | 14.07 | 0 | 8 | 12 | 0.85 | 1.55 | 2.49 | 0.1555 |
| 195 | 38 | 14.07 | 0 | 4 | 12 | 0.85 | 1.55 | 2.49 | 0 |
| 196 | 37.7 | 14.07 | 30 | 0 | 12 | 0.45 | 0.93 | 1.49 | 18.92 |
| 197 | 37.7 | 14.07 | 35 | 0 | 12 | 0.45 | 0.93 | 1.49 | 19.46 |
| 198 | 37.7 | 14.07 | 40 | 0 | 12 | 0.45 | 0.93 | 1.49 | 22.03 |
| 199 | 37.7 | 14.07 | 50 | 0 | 12 | 0.45 | 0.93 | 1.49 | 23.48 |
| 200 | 37.7 | 14.07 | 25 | 0 | 12 | 0.65 | 1.34 | 1.49 | 17.25 |
| 201 | 37.7 | 14.07 | 30 | 0 | 12 | 0.65 | 1.34 | 1.49 | 18.98 |
| 202 | 37.7 | 14.07 | 35 | 0 | 12 | 0.65 | 1.34 | 1.49 | 20.72 |
| 203 | 37.7 | 14.07 | 40 | 0 | 12 | 0.65 | 1.34 | 1.49 | 22.38 |
| 204 | 37.7 | 14.07 | 50 | 0 | 12 | 0.65 | 1.34 | 1.49 | 24.26 |
| 205 | 37.7 | 14.07 | 25 | 0 | 14.5 | 0.45 | 1.05 | 1.49 | 14 |
| 206 | 37.7 | 14.07 | 30 | 0 | 14.5 | 0.45 | 1.05 | 1.49 | 17.14 |
| 207 | 37.7 | 14.07 | 35 | 0 | 14.5 | 0.45 | 1.05 | 1.49 | 20.41 |
| 208 | 37.7 | 14.07 | 40 | 0 | 14.5 | 0.45 | 1.05 | 1.49 | 21.89 |
| 209 | 37.7 | 14.07 | 50 | 0 | 14.5 | 0.45 | 1.05 | 1.49 | 22.71 |
| 210 | 37.7 | 14.07 | 25 | 0 | 14.5 | 0.65 | 1.52 | 1.49 | 11.46 |
| 211 | 37.7 | 14.07 | 30 | 0 | 14.5 | 0.65 | 1.52 | 1.49 | 14.33 |
| 212 | 37.7 | 14.07 | 35 | 0 | 14.5 | 0.65 | 1.52 | 1.49 | 16.58 |
| 213 | 37.7 | 14.07 | 40 | 0 | 14.5 | 0.65 | 1.52 | 1.49 | 18.08 |
| 214 | 37.7 | 14.07 | 50 | 0 | 14.5 | 0.65 | 1.52 | 1.49 | 18.64 |
| 215 | 37.7 | 14.07 | 20 | 0 | 10 | 0.85 | 1.54 | 1.49 | 11.58 |
| 216 | 37.7 | 14.07 | 20 | 0 | 10 | 0.85 | 1.54 | 1.52 | 11.61 |
| 217 | 37.7 | 14.07 | 20 | 0 | 10 | 0.85 | 1.54 | 1.56 | 12.16 |
| 218 | 37.7 | 14.07 | 20 | 0 | 10 | 0.85 | 1.54 | 1.6 | 12 |
| 219 | 37.7 | 14.07 | 20 | 0 | 10 | 0.85 | 1.54 | 1.64 | 12.47 |
| 220 | 37.7 | 14.07 | 20 | 0 | 10 | 0.85 | 1.54 | 1.68 | 12.62 |
| 221 | 37.7 | 14.07 | 20 | 0 | 10 | 0.85 | 1.54 | 1.8 | 13.05 |
| 222 | 37.7 | 14.07 | 20 | 0 | 10 | 0.85 | 1.54 | 1.91 | 13.37 |
| 223 | 37.7 | 14.07 | 20 | 0 | 10 | 0.85 | 1.54 | 1.99 | 14.32 |
| 224 | 37.7 | 14.07 | 20 | 0 | 10 | 0.85 | 1.54 | 2.06 | 15.57 |
| 225 | 37.7 | 14.07 | 20 | 0 | 10 | 0.85 | 1.54 | 2.14 | 17.55 |
| 226 | 37.7 | 14.07 | 20 | 0 | 10 | 0.85 | 1.54 | 2.22 | 18.33 |
| 227 | 37.7 | 14.07 | 20 | 0 | 10 | 0.85 | 1.54 | 2.26 | 18.31 |
| 228 | 37.7 | 14.07 | 20 | 0 | 12 | 0.65 | 1.34 | 1.49 | 12.35 |
| 229 | 37.7 | 14.07 | 20 | 0 | 12 | 0.65 | 1.34 | 1.52 | 13.03 |
| 230 | 37.7 | 14.07 | 20 | 0 | 12 | 0.65 | 1.34 | 1.55 | 13.6 |
| 231 | 37.7 | 14.07 | 20 | 0 | 12 | 0.65 | 1.34 | 1.58 | 14.24 |
| 232 | 37.7 | 14.07 | 20 | 0 | 12 | 0.65 | 1.34 | 1.62 | 14.7 |
| 233 | 37.7 | 14.07 | 20 | 0 | 12 | 0.65 | 1.34 | 1.65 | 15.01 |
| 234 | 37.7 | 14.07 | 20 | 0 | 12 | 0.65 | 1.34 | 1.75 | 14.93 |
| 235 | 37.7 | 14.07 | 20 | 0 | 12 | 0.65 | 1.34 | 1.85 | 15.27 |
| 236 | 37.7 | 14.07 | 20 | 0 | 12 | 0.65 | 1.34 | 1.92 | 14.72 |
| 237 | 37.7 | 14.07 | 20 | 0 | 12 | 0.65 | 1.34 | 1.99 | 13.38 |
| 238 | 37.7 | 14.07 | 20 | 0 | 14.5 | 0.65 | 1.52 | 1.49 | 12.57 |
| 239 | 37.7 | 14.07 | 20 | 0 | 14.5 | 0.65 | 1.52 | 1.52 | 13.54 |
| 240 | 37.7 | 14.07 | 20 | 0 | 14.5 | 0.65 | 1.52 | 1.56 | 13.38 |
| 241 | 37.7 | 14.07 | 20 | 0 | 14.5 | 0.65 | 1.52 | 1.6 | 13.73 |
| 242 | 38 | 14.07 | 20 | 0 | 12 | 0.85 | 1.75 | 1.49 | 10.042 |
| 243 | 38 | 14.07 | 16 | 0 | 12 | 0.85 | 1.75 | 1.49 | 9.6476 |
| 244 | 38 | 14.07 | 12 | 0 | 12 | 0.85 | 1.75 | 1.49 | 8.2621 |
| 245 | 38 | 14.07 | 8 | 0 | 12 | 0.85 | 1.75 | 1.49 | 0.1486 |
| 246 | 38 | 14.07 | 4 | 0 | 12 | 0.85 | 1.75 | 1.49 | 0.0337 |
| 247 | 38 | 14.07 | 20 | 0 | 15 | 0.45 | 1.05 | 1.49 | 10.809 |
| 248 | 38 | 14.07 | 16 | 0 | 15 | 0.45 | 1.05 | 1.49 | 9.8781 |
| 249 | 38 | 14.07 | 12 | 0 | 15 | 0.45 | 1.05 | 1.49 | 0.2692 |
| 250 | 38 | 14.07 | 8 | 0 | 15 | 0.45 | 1.05 | 1.49 | 0.1788 |
| 251 | 38 | 14.07 | 4 | 0 | 15 | 0.45 | 1.05 | 1.49 | 0.0567 |
| 252 | 38 | 14.07 | 20 | 0 | 15 | 0.65 | 1.52 | 1.49 | 11.033 |
| 253 | 38 | 14.07 | 16 | 0 | 15 | 0.65 | 1.52 | 1.49 | 8.187 |
| 254 | 38 | 14.07 | 12 | 0 | 15 | 0.65 | 1.52 | 1.49 | 3.5342 |
| 255 | 38 | 14.07 | 8 | 0 | 15 | 0.65 | 1.52 | 1.49 | 0.099 |
| 256 | 38 | 14.07 | 4 | 0 | 15 | 0.65 | 1.52 | 1.49 | 0.0382 |
| 257 | 38 | 14.07 | 20 | 0 | 15 | 0.85 | 1.98 | 1.49 | 8.6611 |
| 258 | 38 | 14.07 | 16 | 0 | 15 | 0.85 | 1.98 | 1.49 | 7.9992 |
| 259 | 38 | 14.07 | 12 | 0 | 15 | 0.85 | 1.98 | 1.49 | 7.754 |
| 260 | 38 | 14.07 | 8 | 0 | 15 | 0.85 | 1.98 | 1.49 | 1.2167 |
| 261 | 38 | 14.07 | 4 | 0 | 15 | 0.85 | 1.98 | 1.49 | 0.0708 |
| 262 | 38 | 14.07 | 0 | 20 | 12 | 0.45 | 0.82 | 2.49 | 0.1174 |
| 263 | 38 | 14.07 | 0 | 16 | 12 | 0.45 | 0.82 | 2.49 | 0.1099 |
| 264 | 38 | 14.07 | 0 | 12 | 12 | 0.45 | 0.82 | 2.49 | 0.0849 |
| 265 | 38 | 14.07 | 0 | 8 | 12 | 0.45 | 0.82 | 2.49 | 0.0169 |
| 266 | 38 | 14.07 | 0 | 4 | 12 | 0.45 | 0.82 | 2.49 | 0 |
| 267 | 38 | 14.07 | 0 | 20 | 12 | 0.65 | 1.18 | 2.49 | 0.2107 |
| 268 | 38 | 14.07 | 0 | 16 | 12 | 0.65 | 1.18 | 2.49 | 0.197 |
| 269 | 38 | 14.07 | 0 | 12 | 12 | 0.65 | 1.18 | 2.49 | 0.1689 |
| 270 | 38 | 14.07 | 0 | 8 | 12 | 0.65 | 1.18 | 2.49 | 0.1103 |
